# The Family Relation

**Published:** 1866-11

**Authors:** 


HALL'S JOURNAL OF HEALTH.

VOL. XIII.] NOVEMBER, 1866. [No. 11.

### THE FAMILY RELATION.

"He was always allowed to have his own way," was the record a few days ago, of a well-dressed young man who was found dead in the road, with a bullet hole through the back of his head. He was the only son of a wealthy widow, who furnished him with all the money he wanted, so that he might enjoy himself; with the result that he fell into bad company, and dissipated habits, and evil associations, and was murdered on the distant shores of California for the jewelry which he had on his person; leaving his mother in all her lonliness in her New England home, to pass the remainder of her life in bitter remorse for having acted on the mistaken principle, that the best way to have a child enjoy himself, was to allow him "to have his own way." Within a day or two, a murderer who had some education, and had enjoyed the advantages of good society, made the deliberate statement under the gallows:

"And now I want to say to all these people to respect their parents. When we throw all parental authority aside we commence a course of sin. It is terrible to be disobedient to parents Sabbath breaking and lying, too, are awful things. There are many here who have children, I want to state to them that it is their great duty to God and their children to guard them well in their youth, and give them good influences to surround them; to take them more into their confidence, and make home inviting and happy to them, and talk to them more. My father was a church member, aud so was my mother; but they never gave me any advice. They went to their church every Sunday, but they left their religion at the church. They never explained to us the doctrines of the Bible. All parents should see that their children have a love for God, and they should let them know what the bible is. A great many children are running around in the streets, and they get them into the jails and that only makes them worse. We should make home happy, and a blessng for them. It is a duty that God devolves upon us. I hope God may pardon all our sins, and that he may receive my soul."

Such facts show that it is the houshold education which moulds the character, and that if the family relation was properly understood and its duties and obligations were properly met, a vast amount of crime, and destitution, and wretchedness, and premature death would be prevented, and in view of the subject the following article from the able pen of the Rev. Dr. Joel Parker of Newark, New Jersey, merits a serious and thoughtful consideration.

